# Single Oral Dose Toxicity Study of Pinelliae Rhizoma Aqueous Extract in ICR Mice

**DOI:** 10.5487/TR.2009.25.3.147

**Published:** 2009-09-01

**Authors:** Young-Kwon Lim, Ji-Ha Park, Bu-ll Seo, Seong-Soo Roh, Sae-Kwang Ku

**Affiliations:** 16 Department of Herbalogy, College of Oriental Medicine, Daegu Haany University, Gyeongsan, 712-715 Korea; 26 Department of Anatomy and Histology, College of Oriental Medicine, Daegu Haany University, 290, Yugok-dong, Gyeongsan-si, Gyeongsangbuk-do, 712-715 Korea; 36 Development Team for The New Drug of Oriental Medicine (BK21 program), Daegu Haany University, Gyeongsan, 712-715 Korea

**Keywords:** Pinelliae Rhizoma, *Pinellia ternata*, Single oral dose toxicity, Mice, Histopathology

## Abstract

This study was conducted to obtain acute information of the oral dose toxicity of lyophilized water extract of Pinelliae Rhizoma, a dried tuber of *Pinellia ternata* (PR) in male and female mice. In order to calculated 50% lethal dose (LD_50_) and approximate lethal dose (ALD), test material was once orally administered to male and female ICR mice at dose levels of 2000, 1000, 500, 250, 125 and 0 (vehicle control) ml/kg (body weight). The mortality and changes in body weight, clinical signs, gross observation, organ weight and histopathology of principle organs were monitored 14 days after treatment with PR extract. We could not find any mortalities, clinical signs, changes in the body and organ weights, gross and histopathological findings except for dose-dependent increases in the hepatic fatty change frequencies detected in PR extract 2000 and 1000mg/kg treated in both male and female mice. The results obtained in this study suggest that LD_50_ and approximate LD in mice after single oral dose of PR extracts were considered over 2000 mg/kg in both and female male mice, but more than 1000mg/kg of PR extracts treatment could induce slight hepatotoxicity the fatty changes in mice.

## INTRODUCTION

A traditional Korean herbal medicine, Pinelliae Rhizoma (PR) is a dried tuber of *Pinellia ternata* (Thunberg) Breitenbach (Family: Araceae), and has been used as sedative and antiemesis agents for pregnant (Ho and Liu, 1975; Maki *et al.*, 1987; Lee *et al.*, 1993; Kurata *et al.*, 1998). Until now, various pharmacological effects of PR have been revealed; anti-cancer effect (Li *et al.*, 2007), anti-obesity effect (Kim *et al.*, 2006), emetic effect (Zhao *et al.*, 2005), anti-emetic effect (Kurata *et al.*, 1998) and neuroprotective effect (Shi *et al.*, 1994). In addition, the therapeutic effects on hepatitis B infections (Chen *et al.*, 2005), neuroprotective effect (Koo *et al.*, 2005; Chung *et al.*, 2006), anti-stress effect (Katagiri *et al.*, 2004) and gastric profusion effect (Chen *et al.*, 2002) of mixed herbal formula containing PR extracts also have been researched.

As increase of the concern in the functional food and well being in life, the demands and consumption of functional food originated from natural sources are increased (Lee *et al.*, 2003). However, the toxicological aspects about these natural origin-functional foods has been neglected because of the reasons that they has been used as various purpose for long times. Therefore, it is considered that more detail and systemic toxicological studies should be tested for control the abuse and potential toxicities even if they have been used as traditional folk medicine. Although it has been regarded that PR is a representative toxic irritable herbs in Korean medicine (Park and Seo, 2000), the scientific toxicological studies about PR extracts have not conducted except for 4 weeks repeated oral dose toxicity test in rats (Lee *et al.*, 2003), cytotoxicity (Lee *et al.*, 1993) and some poster presentational gentoxicity (Yang *et al.*, 1990). There are no repots dealing the toxicological aspects of PR water extracts in mouse, even if the basic single dose toxicities.

The objective of the present study, therefore, was to obtain the primary safety information about PR extracts, lyophilized water extract of *Pinellia ternata* and further clarifies their safety for clinical use. In order to observe the 50% lethal dose ($LD_{50}$), approximate lethal dosage (ALD), test articles were once orally administered to female and male ICR mice at dose levels of 2000, 1000, 500 and 0 (control) mg/kg (body weight) according to the recommendation of KFDA Guidelines (2005-60, 2005). The mortality and changes on body weight, clinical signs and gross observation were monitored during 14 days after oral administration of PR extracts according to Korea Food and Drug Administration (KFDA) Guidelines (2005-60, 2005) with organ weights and histopathology of 12 types of principle organs.

## MATERIALS AND METHODS

***Experimental animals.*** Each of twenty female and male ICR mice (6-wk old upon receipt, SLC, Japan) was used after acclimatization for 7 days. Five animals were allocated per a polycarbonate cage in a temperature (20~25°C) and humidity (40~45%) controlled room. Light : dark cycle was 12 h : 12 h and feed (Samyang, Korea) and water were supplied free to access. All animals were overnight fasted (about 18 h) before dosing and terminal necropsy. Animals were marked by picric acid.

***Preparation of PR extracts.*** Aqueous PR extracts (absorption rate 29.36%) were prepared by routine methods using rotary vacuum evaporator (BUCHI Rotavapor R-144, Switzerland) and programmable freeze dryer (IlShin Lab., Korea) from PR, which were purchased from local voucher (Cho-Heung Pharmaceutical Ind. Co. (Daegu, Korea) after confirm the morphology under microscopy. Powders of PR extracts were light brown powder. PR extracts were stored in a refrigerator at -20°C to protect from light and degeneration. The appearance of PR extracts in vehicle was clear light brown solution in distilled water and it is well soluble upto 200 mg/ml concentration levels. The test article was single orally administered at a dosage volume of 20 ml/kg using distilled water as vehicle.

***Grouping and dosing.*** The animals were distributed into 8 groups 5 mice per group upon receipt. PR extracts have been used as folk medicine and ingredients of medicinal food for long times and no revealed toxicological data was available, the highest dosage level was selected as 2000 mg/kg according to the recommended by KFDA (2005-60, 2005) and Organization for Economic Co-Operation and Development (OECD) (2001) guidelines, the limited dosages, and 1000 and 500 mg/kg was selected using common ratio 2. In addition, a vehicle control group was added. Animal was once orally dosed using a sonde attached to a syringe of 1 ml after overnight fasting (about 18hr, water was not restricted). Feed and water were restricted further for about 3 h.

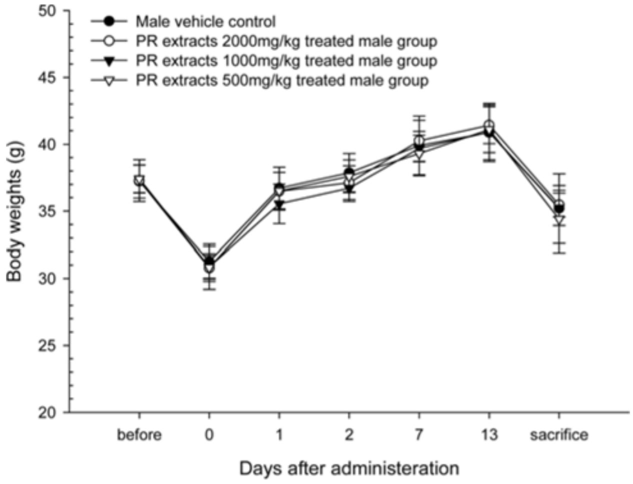

**Fig. 1.** Body weight changes in male mice after once oral dose of PR extract. No meaningful changes were detected in all PR extract treated groups as compared with vehicle control. Before means 1 day before administration; Day 0 means at administration; All animals at sacrifice and Day 0 overnight fasted.

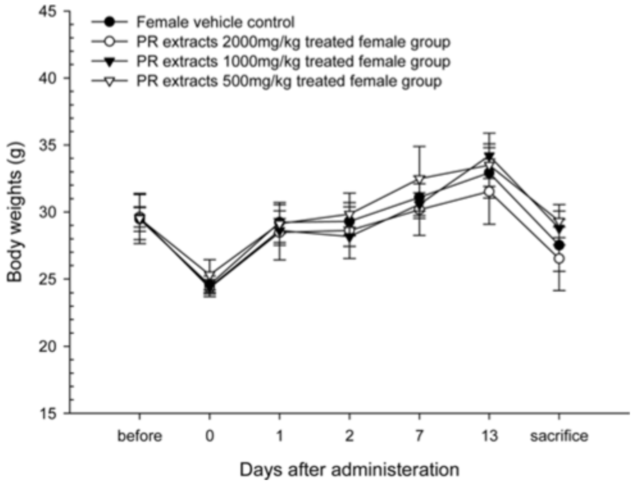

**Fig. 2.** Body weight changes in female mice after single oral dose of PR extract. No meaningful changes were detected in all PR extract treated groups as compared with vehicle control. Before means 1 day before administration; Day 0 means at administration; All animals at sacrifice and Day 0 overnight fasted.

***Observation of clinical signs.*** All abnormal clinical signs were recorded before and after dosing at least twice a day based on the functional observational battery test (Irwin, 1968; Dourish, 1987).

***Body weight changes.*** Body weights were measured at the day of dosing (Day 0) immediately before treatment, 1, 2, 7, 13 and 14 days after dosing. In addition, to reduce the individual body weight differences of animals at initial dosing, body weigh gains during Day 0~Day 7, Day 7~Day 13 and Day 0~Day 14 were also calculated based on measured body weight at each day.

***Necropsy.*** All unscheduled died animals were grossly observed immediately after finding them and all survived animals were subjected to terminal necropsy. Animals were asphyxiated by carbon dioxide and gross necropsy was performed in all animals at Day 14 after overnight fasting (about 18 h, water was not restricted).

Specific organs grossly observed: lung, heart, kidney, spleen, testis, liver, pancreas, epididymis, popliteal lymph node, ovary, brain, and uterus.

***Organ weight measurement.*** The absolute organ weight was measured and then relative organ weight (% of body weight) was calculated for the following organs of all experimental animals when they were sacrificed.

Measured organs: lung, heart, kidney (left), spleen, testis (left), liver, pancreas (splenic lobes), epididymis (left), popliteal lymph node (left), ovary (left), brain, and uterus.

***Histopathology.*** Principle organs listed below were sampled at terminal necropsy, and fixed in 10% NBF (neutral buffered formalin). After 18 h of fixation, paraffin embedding was conducted and 3~4 μm sections were

**Table 1.** Body weight (g) gains after oral treatment of PR extracts

| Group | Intervals | | |
|---|---|---|---|
| | Day 0[a]~Day 7 | Day 7~Day 13 | Day 0~Day 14 |
| Male | | | |
| Vehicle control | 8.64 ± 1.25 | 0.98 ± 0.84 | 3.94 ± 1.83 |
| 2000 mg/kg | 9.48 ± 1.75 | 1.16 ± 0.85 | 4.68 ± 1.07 |
| 1000 mg/kg | 8.82 ± 0.78 | 1.22 ± 1.32 | 4.38 ± 1.08 |
| 500 mg/kg | 8.48 ± 1.26 | 1.80 ± 0.51 | 3.58 ± 2.24 |
| Female | | | |
| Vehicle control | 3.90 ± 1.83 | 1.76 ± 0.85 | 2.96 ± 2.07 |
| 2000 mg/kg | 3.24 ± 1.57 | 2.28 ± 1.40 | 3.50 ± 1.07 |
| 1000 mg/kg | 4.32 ± 0.43 | 1.24 ± 0.59 | 2.14 ± 1.15 |
| 500 mg/kg | 5.26 ± 1.82 | 0.96 ± 1.63 | 3.72 ± 1.22 |

Values are expressed as mean ± S.D., g (n = 5); [a]Day of treatment.

**Table 2.** Changes on the absolute organ weights (g) after oral treatment of PR extracts

| Group | Organs: Male | | | | | | | | | | | |
|---|---|---|---|---|---|---|---|---|---|---|---|---|
| | Lung | Heart | Thymus | Kidney L | Adrenal gland L | Spleen | Testis L | Liver | Pancreas S | Brain | Epididymis L | Lymph node L[a] |
| Vehicle control | 0.215 ± 0.007 | 0.179 ± 0.011 | 0.062 ± 0.013 | 0.307 ± 0.048 | 0.008 ± 0.002 | 0.113 ± 0.027 | 0.127 ± 0.013 | 1.777 ± 0.179 | 0.185 ± 0.027 | 0.484 ± 0.017 | 0.060 ± 0.012 | 0.012 ± 0.003 |
| 2000 mg/kg | 0.213 ± 0.022 | 0.178 ± 0.013 | 0.058 ± 0.006 | 0.305 ± 0.031 | 0.006 ± 0.002 | 0.117 ± 0.009 | 0.127 ± 0.017 | 1.839 ± 0.108 | 0.215 ± 0.032 | 0.494 ± 0.017 | 0.049 ± 0.004 | 0.009 ± 0.004 |
| 1000 mg/kg | 0.210 ± 0.015 | 0.187 ± 0.014 | 0.052 ± 0.012 | 0.322 ± 0.022 | 0.008 ± 0.004 | 0.103 ± 0.015 | 0.118 ± 0.012 | 1.833 ± 0.096 | 0.193 ± 0.019 | 0.487 ± 0.013 | 0.047 ± 0.007 | 0.012 ± 0.003 |
| 500 mg/kg | 0.208 ± 0.021 | 0.183 ± 0.015 | 0.033 ± 0.016* | 0.328 ± 0.051 | 0.005 ± 0.004 | 0.088 ± 0.013 | 0.123 ± 0.011 | 1.788 ± 0.346 | 0.176 ± 0.019 | 0.482 ± 0.015 | 0.049 ± 0.006 | 0.011 ± 0.004 |
| | Organs: Female | | | | | | | | | | | |
| | Lung | Heart | Thymus | Kidney L | Adrenal gland L | Spleen | Ovary L | Liver | Pancreas S | Brain | Uterus | Lymph node L |
| Vehicle control | 0.193 ± 0.009 | 0.149 ± 0.011 | 0.059 ± 0.008 | 0.211 ± 0.020 | 0.007 ± 0.003 | 0.097 ± 0.017 | 0.023 ± 0.006 | 1.312 ± 0.149 | 0.165 ± 0.013 | 0.484 ± 0.021 | 0.205 ± 0.027 | 0.013 ± 0.002 |
| 2000 mg/kg | 0.192 ± 0.017 | 0.144 ± 0.012 | 0.055 ± 0.012 | 0.205 ± 0.014 | 0.006 ± 0.003 | 0.098 ± 0.039 | 0.021 ± 0.004 | 1.259 ± 0.163 | 0.151 ± 0.027 | 0.472 ± 0.012 | 0.161 ± 0.056 | 0.011 ± 0.005 |
| 1000 mg/kg | 0.198 ± 0.026 | 0.154 ± 0.009 | 0.067 ± 0.011 | 0.220 ± 0.014 | 0.008 ± 0.004 | 0.122 ± 0.036 | 0.024 ± 0.009 | 1.517 ± 0.132 | 0.184 ± 0.022 | 0.469 ± 0.016 | 0.204 ± 0.047 | 0.011 ± 0.002 |
| 500 mg/kg | 0.192 ± 0.015 | 0.145 ± 0.016 | 0.061 ± 0.010 | 0.208 ± 0.031 | 0.007 ± 0.004 | 0.114 ± 0.013 | 0.028 ± 0.005 | 1.386 ± 0.163 | 0.175 ± 0.021 | 0.470 ± 0.019 | 0.152 ± 0.050 | 0.011 ± 0.003 |

Values are expressed as mean ± S.D., organ weight (g) (n = 5); L, left sides; S, splenic lobes; [a]Popliteal lymph node; * $p < 0.05$ as compared with vehicle control.

prepared by routine histological methods. Representative sections of each specified organs were stained with Hematoxylin & Eosin for light microscopical examination.

Specific organs sampled: lung, heart, kidney (left), spleen, testis (left), liver, pancreas (splenic lobes), epididymis (left), popliteal lymph node (left), ovary (left),

**Table 3.** Changes on the relative organ weights (g) after oral treatment of PR extracts

| Group | Organs: Male | | | | | | | | | | | |
|---|---|---|---|---|---|---|---|---|---|---|---|---|
| | Lung | Heart | Thymus | Kidney L | Adrenal gland L | Spleen | Testis L | Liver | Pancreas S | Brain | Epididymis L | Lymph node L[a] |
| Vehicle control | 0.193 ± 0.009 | 0.149 ± 0.011 | 0.059 ± 0.008 | 0.211 ± 0.020 | 0.007 ± 0.003 | 0.097 ± 0.017 | 0.023 ± 0.006 | 1.312 ± 0.149 | 0.165 ± 0.013 | 0.484 ± 0.021 | 0.205 ± 0.027 | 0.013 ± 0.002 |
| 2000 mg/kg | 0.192 ± 0.017 | 0.144 ± 0.012 | 0.055 ± 0.012 | 0.205 ± 0.014 | 0.006 ± 0.003 | 0.098 ± 0.039 | 0.021 ± 0.004 | 1.259 ± 0.163 | 0.151 ± 0.027 | 0.472 ± 0.012 | 0.161 ± 0.056 | 0.011 ± 0.005 |
| 1000 mg/kg | 0.198 ± 0.026 | 0.154 ± 0.009 | 0.067 ± 0.011 | 0.220 ± 0.014 | 0.008 ± 0.004 | 0.122 ± 0.036 | 0.024 ± 0.009 | 1.517 ± 0.132 | 0.184 ± 0.022 | 0.469 ± 0.016 | 0.204 ± 0.047 | 0.011 ± 0.002 |
| 500 mg/kg | 0.192 ± 0.015 | 0.145 ± 0.016 | 0.061 ± 0.010 | 0.208 ± 0.031 | 0.007 ± 0.004 | 0.114 ± 0.013 | 0.028 ± 0.005 | 1.386 ± 0.163 | 0.175 ± 0.021 | 0.470 ± 0.019 | 0.152 ± 0.050 | 0.011 ± 0.003 |
| | Organs: Female | | | | | | | | | | | |
| | Lung | Heart | Thymus | Kidney L | Adrenal gland L | Spleen | Ovary L | Liver | Pancreas S | Brain | Uterus | Lymph node L |
| Vehicle control | 0.705 ± 0.050 | 0.543 ± 0.059 | 0.216 ± 0.020 | 0.768 ± 0.051 | 0.025 ± 0.008 | 0.350 ± 0.054 | 0.085 ± 0.016 | 4.759 ± 0.316 | 0.599 ± 0.023 | 1.765 ± 0.112 | 0.744 ± 0.245 | 0.047 ± 0.009 |
| 2000 mg/kg | 0.727 ± 0.042 | 0.543 ± 0.031 | 0.205 ± 0.037 | 0.773 ± 0.039 | 0.022 ± 0.009 | 0.365 ± 0.117 | 0.079 ± 0.016 | 4.744 ± 0.359 | 0.569 ± 0.088 | 1.791 ± 0.138 | 0.607 ± 0.198 | 0.042 ± 0.022 |
| 1000 mg/kg | 0.685 ± 0.068 | 0.435 ± 0.038 | 0.234 ± 0.042 | 0.762 ± 0.031 | 0.027 ± 0.015 | 0.420 ± 0.112 | 0.083 ± 0.027 | 5.263 ± 0.332 | 0.642 ± 0.095 | 1.630 ± 0.052 | 0.708 ± 0.167 | 0.040 ± 0.009 |
| 500 mg/kg | 0.653 ± 0.031 | 0.494 ± 0.037 | 0.209 ± 0.041 | 0.707 ± 0.038 | 0.022 ± 0.014 | 0.390 ± 0.049 | 0.095 ± 0.019 | 4.721 ± 0.439 | 0.595 ± 0.050 | 1.603 ± 0.044* | 0.525 ± 0.195 | 0.038 ± 0.008 |

Values are expressed as mean ± S.D., % of body weight at sacrifice (n = 5); L, left sides; S, splenic lobes; [a]Popliteal lymph node; * $p < 0.05$ as compared with vehicle control.

**Table 4.** Necropsy findings after oral treatment of PR extracts

| Group | Male | | | | Female | | | |
|---|---|---|---|---|---|---|---|---|
| | Vehicle control | 2000 mg/kg | 1000 mg/kg | 500 mg/kg | Vehicle control | 2000 mg/kg | 1000 mg/kg | 500 mg/kg |
| Lung | | | | | | | | |
| Normal | 5/5 | 5/5 | 5/5 | 5/5 | 4/5 | 4/5 | 4/5 | 5/5 |
| Congestion | 0/5 | 0/5 | 0/5 | 0/5 | 1/5 | 1/5 | 1/5 | 0/5 |
| Thymus | | | | | | | | |
| Normal | 4/5 | 5/5 | 4/5 | 4/5 | 5/5 | 5/5 | 5/5 | 5/5 |
| Atrophy | 1/5 | 0/5 | 1/5 | 1/5 | 0/5 | 0/5 | 0/5 | 0/5 |
| Spleen | | | | | | | | |
| Normal | 4/5 | 5/5 | 3/5 | 4/5 | 4/5 | 2/5 | 4/5 | 4/5 |
| Atrophy | 0/5 | 0/5 | 2/5 | 1/5 | 0/5 | 2/5 | 1/5 | 0/5 |
| Hypertrophy | 1/5 | 0/5 | 0/5 | 0/5 | 1/5 | 1/5 | 0/5 | 1/5 |
| Liver | | | | | | | | |
| Normal | 5/5 | 5/5 | 5/5 | 4/5 | 5/5 | 5/5 | 5/5 | 5/5 |
| Atypical Foci | 0/5 | 0/5 | 0/5 | 1/5 | 0/5 | 0/5 | 0/5 | 0/5 |
| Epididymis/Uterus | | | | | | | | |
| Normal | 5/5 | 5/5 | 5/5 | 5/5 | 4/5 | 4/5 | 2/5 | 4/5 |
| Edematous changes | 0/5 | 0/5 | 0/5 | 0/5 | 1/5 | 1/5 | 3/5 | 1/5 |
| Lymph node[a] | | | | | | | | |
| Normal | 4/5 | 5/5 | 5/5 | 4/5 | 5/5 | 5/5 | 5/5 | 4/5 |
| Hypertrophy | 1/5 | 0/5 | 0/5 | 1/5 | 0/5 | 0/5 | 0/5 | 1/5 |

Observed animals/total observed animals (n = 5); [a]Bilateral popliteal lymph node.

brain, and uterus.

***Statistical analyses.*** Multiple comparison tests for different dose groups were conducted. Variance homogeneity was examined using the Levene test. If the Levene test indicated no significant deviations from variance homogeneity, the obtain data were analyzed by one way ANOVA test followed by Scheffe test to determine which pairs of group comparison were significantly different. In case of significant deviations from variance homogeneity were observed at Levene test, a non-parametric comparison test, the Mann-Whitney U-Wilcoxon Rank Sum W test was conducted to determine the specific pairs of group comparison, which are significantly different. $LD_{50}$ and 95% confidence limits were calculated by Probit method. Statistical analyses were conducted using SPSS for Windows (Release 14.0 K, SPSS Inc., USA) and a *p*-value of less than 0.05 was considered to be a significant difference. In addition, degree of clinical signs, gross and histopathological findings were subdivided into 3 degrees: 3+ Severe, 2+ moderate, 1+ slight.

## RESULTS

***Mortalities.*** No unscheduled or PR extract-treat related mortalities were detected in all dose levels

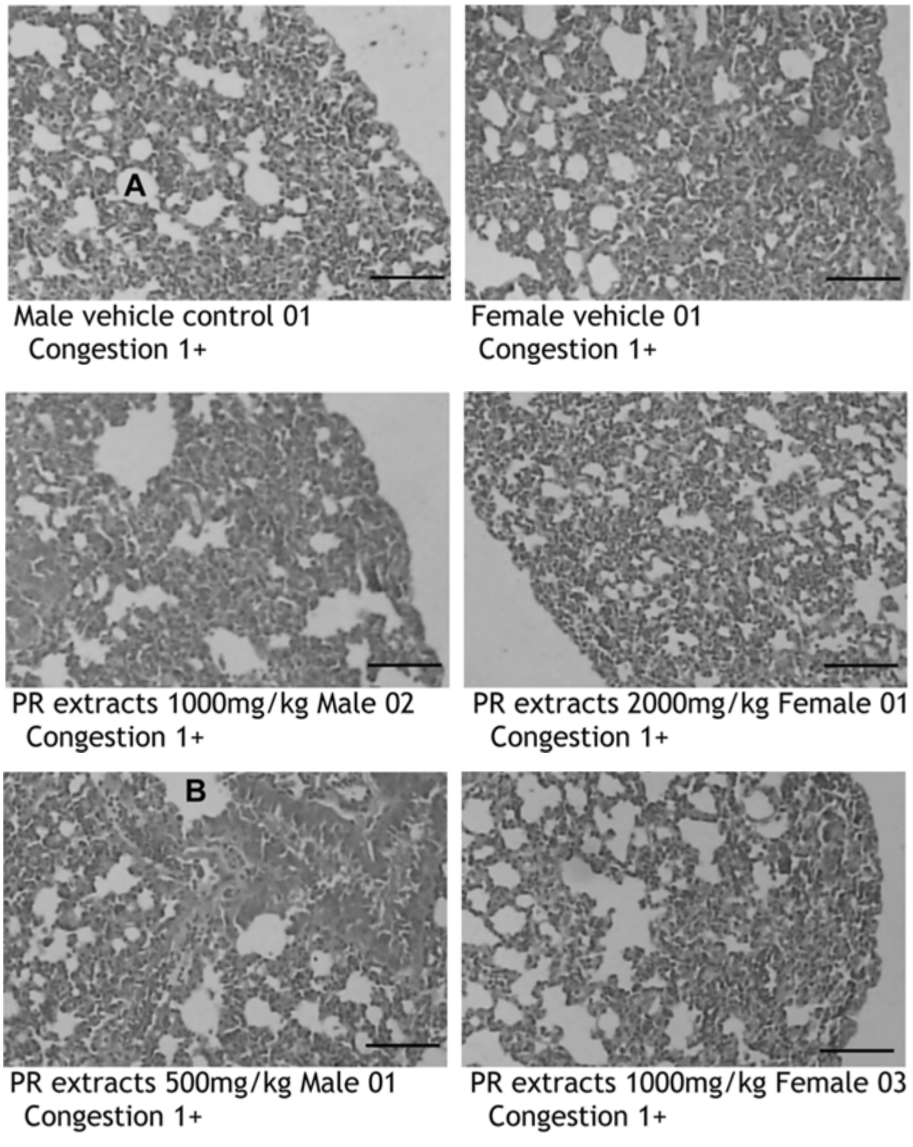

**Fig. 3.** Histopathological changes detected on the lung. Note that hypertrophy of alveolus with hemorrhages were randomly detected dispersed throughout the all tested groups including vehicle controls as an accidental findings. They did not show any dose dependency. A, alveolus; B, bronchiole; All Hematoxylin & Eosin stain; Scale bars = 160 μm.

tested in this study. At terminal, all of animals (5/5; 100%) were survived in all dose levels tested including vehicle control.

***Clinical signs.*** In this study, no PR extracts-treatment related abnormal clinical signs were observed during observation periods regardless of male and female mice.

***Changes in body weights and gains.*** No meaningful changes on body weight and gains were detected in all dosing groups tested compared to that of vehicle control in all dose levels tested (Fig. 1 and 2, Table 1).

***Changes in the organ weight.*** No meaningful changes on the absolute and relative organ weight of 12 principle organs were observed in all dosing groups tested compared to that of vehicle control except for significantly ($p < 0.05$) increases of absolute and relative weight of thymus in PR extract 500mg/kg-dosing male group, and significantly ($p < 0.05$) increase of relative brain weight in PR extracts 500mg/kg-dosing female group as compared with each gender of vehicle control, respectively (Table 2 and 3).

***Necropsy findings.*** No meaningful changes on the gross findings of 12 principle organs were observed in all dosing groups tested compared to that of vehicle control except for some accidental findings such as congestion spots of lung, atrophy of thymus, spleen atrophy or hypertrophy, hypertrophy of popliteal lymph node, edematous changes of uterus, and hypertrophy of popliteal lymph nodes. They were randomly detected throughout the whole experimental groups including each gender of vehicle controls, and most of these sporadic gross findings do not showed dose-dependent frequencies encountered. Atypical white-yellow focal foci in liver were restrictly detected in one mouse of PR extracts 500mg/kg treated male (Table 4).

***Histopathological findings.*** No meaningful changes in the histopathological findings of 12 principle organs were observed in all dosing groups tested compared to that of vehicle control except for some sporadic acci-

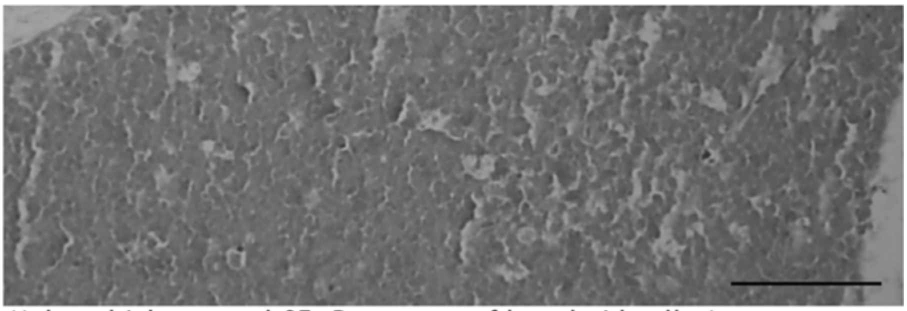

Male vehicle control 05: Decreases of lymphoid cells 1+

**Fig. 4.** Histopathological changes detected in the thymus. Note that decreases of lymphoid cells in the cortex (as seen vacuole) was restricted to one animal (1/5; 20%) of male vehicle control as individual sporadic findings; All Hematoxylin & Eosin stain; Scale bars = 160 μm.

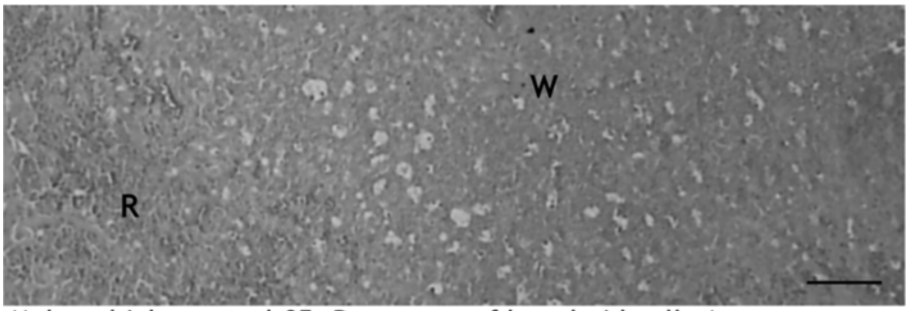

Male vehicle control 05: Decreases of lymphoid cells 1+

**Fig. 5.** Histopathological changes detected in the spleen. Note that decreases of lymphocytes in the white pulp (as seen vacuole) of spleen was restricted to one animal (1/5; 20%) of female vehicle control as individual sporadic findings. R, red pulp; W, white pulp; All Hematoxylin & Eosin stain; Scale bars = 160 μm.

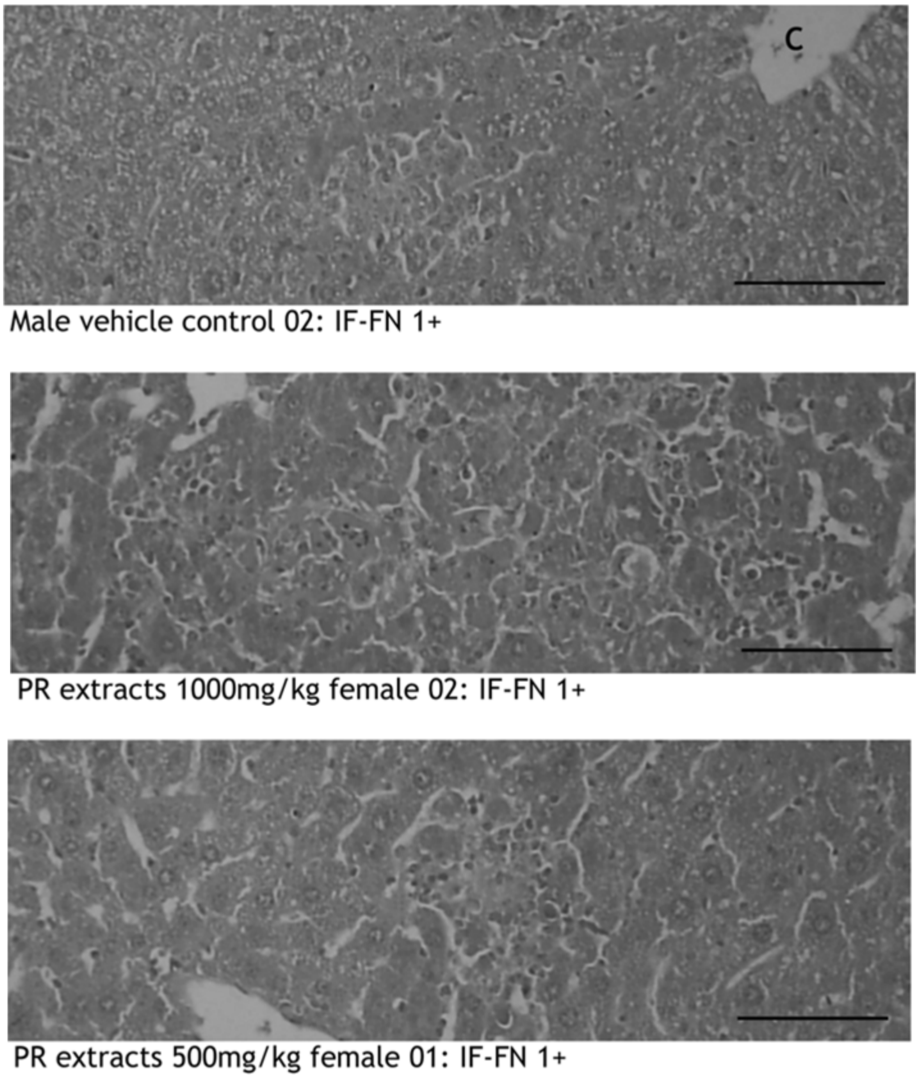

**Fig. 6.** Histopathological changes detected in the liver. Note that focal necrosis and inflammatory cell infiltration (IF-FN) in liver was restrictly observed in one mouse (1/5; 20%) of male vehicle control, female 1000 and 500 mg/kg treated groups, respectively as an accidental findings. They were deposited between hepatic cords or as like islets. C, central vein; All Hematoxylin & Eosin stain; Scale bars = 160 μm.

dental findings such as hypertrophy of lung alveolus wall as congestion (Fig. 3), depletion of lymphoid cells in the cortex of thymus (Fig. 4) and white pulp of spleen (Fig. 5), focal necrosis and inflammatory cell infiltration in liver (Fig. 6), desquamation of the uterus mucosa (Fig. 7). They were randomly detected throughout the whole experimental groups including each gender of vehicle controls, and most of these sporadic histopathological findings do not show dose-dependent frequencies encounted. In addition, somewhat increases in frequency of liver fatty changes were detected in PR extracts 2000 and 1000 mg/kg treated male and female groups (Fig. 8); only one mouse in male and female vehicle control showed slight (1+) hepatic fatty changes but four to two mice in PR extracts 2000 and 1000 mg/kg treated male and female groups showed slight (1+) hepatic fatty changes with one mice in PR extracts 500mg/kg treated groups (Table 5).

## DISCUSSION

In the present study, we investigated the acute toxicity of single oral dose with PR extracts, lyophilized water extract of dried tuber of *Pinellia temata* to female and male mice as a part of the safety test. PR extracts were once orally administered to female and male ICR mice at dose levels of 2000, 1000, 500 and 0 mg/kg according to the recommendation of KFDA Guidelines

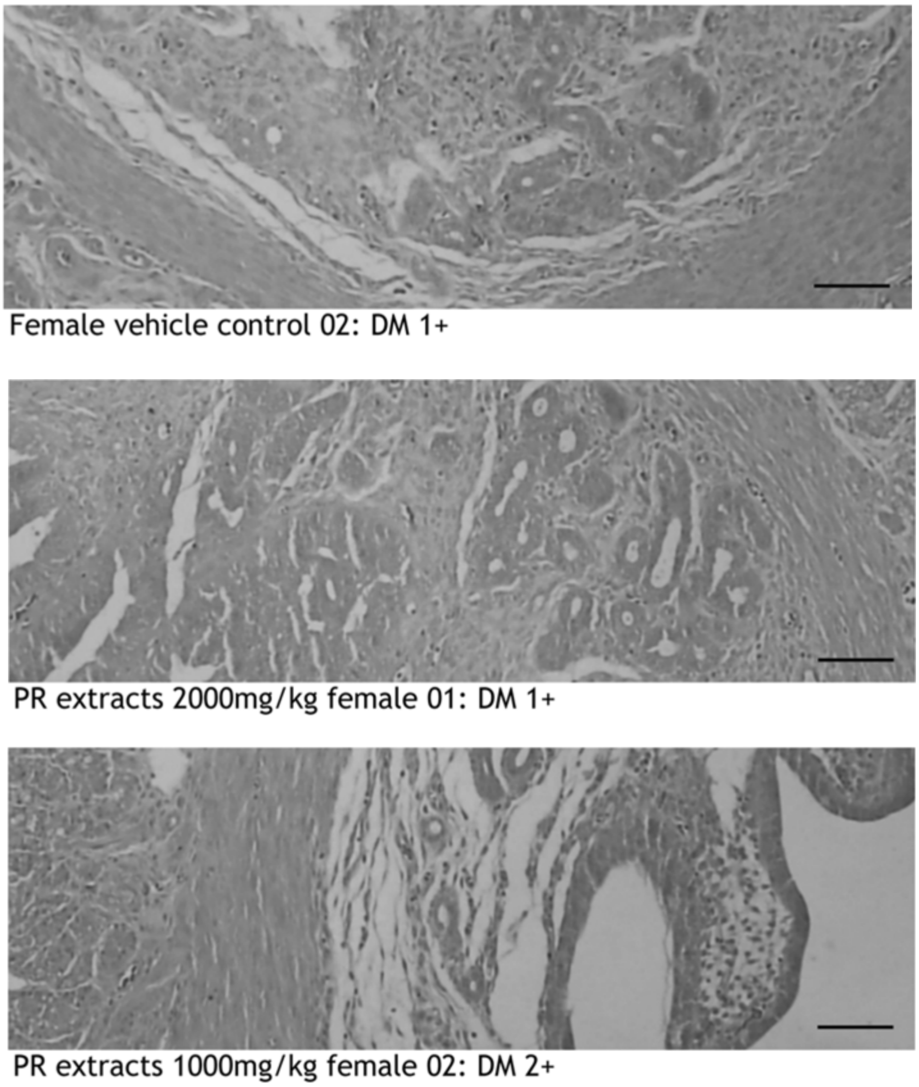

**Fig. 7.** Histopathological changes detected in the uterus. Note that desquamation of the uterus mucosa (DM) were detected as a process of estrus cycle. They were randomly detected throughout the whole female groups including vehicle control. All Hematoxylin & Eosin stain; Scale bars = 160 μm.

(2005-60, 2005). The mortality and changes in body weight, clinical signs and gross observation were monitored during 14 days after treatment of PR extracts with organ weight and histopathology of 12 types of principle organs.

As the results, we could not find any mortality, clinical signs, changes in the body weight and gross findings and some sporadic gross findings. In addition, no meaningful changes in the organ weight and histopathology of 12 types of principle organs were detected in the present study except for dose-dependent increases of frequencies of hepatic fatty changes in PR extracts 2000 and 1000 mg/kg treated both male and female mice, and some sporadic accidental histopathological findings with dose-independent decreases of thymic absolute and relative weights in male 500 mg/kg treated mice, relative brain weights in female 500 mg/kg treated mice, respectively.

The body weight detected in this study was well corresponded to the body weight ranges of same aged normal mice as previously (Plata and Murphy, 1972; Yamaguchi *et al*., 1983) in all tested groups including both male and female vehicle control, respectively. It means PR extracts did not induce any harmful changes on the body weights.

In KFDA Guidelines (2005-60, 2005) and OECD Guidelines (#423, 2001), the recommended highest dose of test materials were 2000 mg/kg or the maximum solubility, and they also recommended that in case of acute toxicity in mice, the dosage volume were

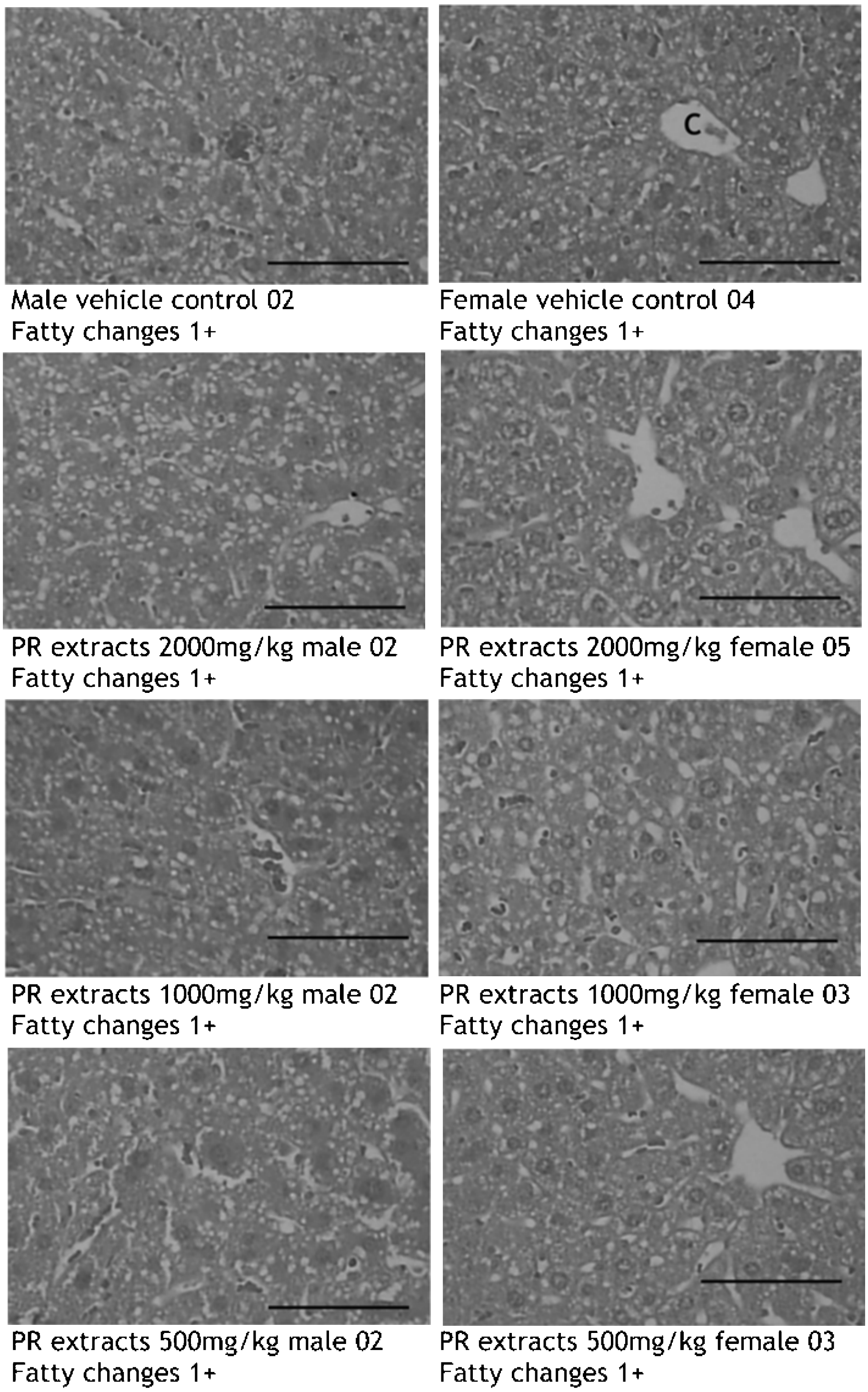

**Fig. 8.** Histopathological changes detected in the liver fatty changes. Note that increases of frequency of liver fatty changes were detected in PR extracts 2000 and 1000 mg/kg treated male and female groups; only one mouse in male and female vehicle control showed slight (1+) fatty changes but they were more frequently detected in PR extracts 2000 and 1000 mg/kg treated male and female groups. They were deposited as lipid droplet in hepatocytes and/or induced hypertrophy of hepatocytes around central veins; C, central vein; All Hematoxylin & Eosin stain; Scale bars = 160 μm.

below 20 ml/kg in case of clear soluble materials but 10 ml/kg in suspensions. In the present study, the highest dose of PR extracts was selected as 2000 mg/kg because PR extracts have been used as folk medicine

**Table 5.** Histopathological findings after oral treatment of PR extracts

| Group | Male | | | | Female | | | |
|---|---|---|---|---|---|---|---|---|
| | Vehicle control | 2000 mg/kg | 1000 mg/kg | 500 mg/kg | Vehicle control | 2000 mg/kg | 1000 mg/kg | 500 mg/kg |
| Lung | | | | | | | | |
| Normal | 4/5 | 5/5 | 4/5 | 3/5 | 4/5 | 4/5 | 4/5 | 5/5 |
| Congestion | 1/5 | 0/5 | 10/5 | 2/5 | 1/5 | 1/5 | 1/5 | 0/5 |
| Thymus | | | | | | | | |
| Normal | 4/5 | 5/5 | 5/5 | 5/5 | 5/5 | 5/5 | 5/5 | 5/5 |
| DE* | 1/5 | 0/5 | 0/5 | 0/5 | 0/5 | 0/5 | 0/5 | 0/5 |
| Spleen | | | | | | | | |
| Normal | 5/5 | 5/5 | 5/5 | 5/5 | 4/5 | 5/5 | 5/5 | 5/5 |
| DE* | 0/5 | 0/5 | 0/5 | 0/5 | 1/5 | 0/5 | 0/5 | 0/5 |
| Liver | | | | | | | | |
| Normal | 4/5 | 1/5 | 2/5 | 4/5 | 4/5 | 2/5 | 3/5 | 3/5 |
| IF-FN* | 1/5 | 0/5 | 0/5 | 0/5 | 0/5 | 0/5 | 1/5 | 1/5 |
| Fatty changes | 1/5 | 4/5 | 3/5 | 1/5 | 1/5 | 3/5 | 1/5 | 1/5 |
| Epididymis/Uterus | | | | | | | | |
| Normal | 5/5 | 5/5 | 5/5 | 5/5 | 3/5 | 4/5 | 2/5 | 5/5 |
| DM* | 0/5 | 0/5 | 0/5 | 0/5 | 2/5 | 1/5 | 3/5 | 0/5 |

Observed animals/total observed animals (n = 5); [a]Bilateral popliteal lymph node; * Abbreviations: DE, decreases of lymphoid cells; IF-FN, inflammatory cell infiltration with necrosis; DM, desquamation of mucosa.

and ingredients of medicinal food for long times and no revealed toxicological data was available, base on the recommendation of KFDA (2005-60, 2005) and OECD Guidelines (#423, 2001), and treated in a volume of 20 ml/kg using distilled water as vehicle because PR extracts were clearly dissolved upto 100mg/ml, at least in the present study.

Significant ($p < 0.05$) decreases of absolute and relative thymic weights in PR extracts 500 mg/kg male mice, and relative brain weights in PR extracts 500mg/kg male mice were considered as not PR extracts-treatment related changes because they were not showed dose-dependency with no changes on the histopathological profiles, and the other organ weights measured in this study well corresponded to the normal mice organ weight ranges as previously (Plata and Murphy, 1972; Yamaguchi *et al.*, 1983).

The depletion of lymphoid cells in thymus detected in one animal of male vehicle control was considered as a result of physiological involution (Banks, 1986), and splenic white pulp lymphoid cells depletion detected in one female vehicle control mouse is also considered as individual changes. Edematous changes in the uterus at gross findings and related desquamation of uterus mucosa at histopathological observation in the present study were also considered as a result of differences of physiological estrus cycles (Banks, 1986; Pineda, 1989) not PR extracts-dosing relative changes. They were also detected in vehicle control. The atypical yellow foci detected in one mouse of PR extract 500 mg/kg treated group revealed as slight fatty changes at histopathological observations.

The dose-dependent increases of frequencies of hepatic fatty changes detected in PR extracts 2000 and 1000 mg/kg treated both male and female mice, considered as direct evidences that PR extracts can be induced hepatotoxicity. The fatty changes in hepatocytes have been regarded as mild damages of liver (Maclachlan and Cullen, 1995). However, in the previous PR extracts 4 weeks repeated oral dose toxicity test in rat (Lee *et al.*, 2003), the possibilities of hepatotoxicity did not suggested. Therefore, it is considered that the toxicity of PR extracts have species-specific patterns.

Congestion spots of lung, atrophy of thymus, spleen atrophy or hypertrophy, hypertrophy of popliteal lymph node and hypertrophy of popliteal lymph nodes detected as gross findings, and hypertrophy of lung alveolus wall as congestion and focal necrosis and inflammatory cell infiltration in liver detected as histopathological findings were considered as accidental findings and they were not considered as PR extracts-treatment related abnormal gross or histopathological findings because they were restricted in some individual animals and most of them, also observed in male and female vehicle controls. . In addition, most of them were rarely observed in normal mice (Lee *et al.*, 2005, 2006).

Although the Hodge and Sterner (1949) classify as non toxic materials those $LD_{50}$ were 5000~15000 mg/kg and the materials those $LD_{50}$ were 500~5000 mg/kg also classified as relatively low toxic (Class III) by US Environmental Protection Agency (OPPTS 870.100,

1998), recently Notified Guidelines by KFDA (2005-60, 2005) and OECD Guidelines (#423, 2001) recommended that the highest oral dose of test materials was 2000 mg/kg. The $LD_{50}$ and ALD in mice after single oral dose of PR extracts were detected over 2000mg/kg in both male and female in the present study. However, more than 1000 mg/kg of PR extracts treatment could be induced slight hepatotoxicity in mice, the fatty changes.
